# Construction of Targeting-Peptide-Based Imaging Reagents and Their Application in Bioimaging

**DOI:** 10.1021/cbmi.3c00104

**Published:** 2023-12-04

**Authors:** Limin Zhang, Xin Wang, Jinge Zhao, Beilei Sun, Weizhi Wang

**Affiliations:** Key Laboratory of Medical Molecule Science and Pharmaceutics Engineering, Ministry of Industry and Information Technology, Key Laboratory of Cluster Science of Ministry of Education, Beijing Key Laboratory of Photoelectronic/Electrophotonic Conversion Materials, School of Chemistry and Chemical Engineering, Institute of Engineering Medicine, Beijing Institute of Technology, Beijing 100081, PR China

**Keywords:** Molecular imaging, Targeting peptide, Self-assembly, Imaging reagents, Peptide screening, OBOC, De novo design, Precise recognition

## Abstract

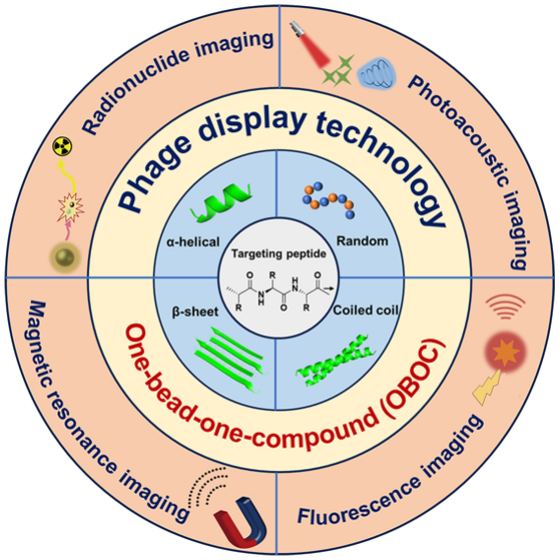

Molecular imaging was developed from basic molecular recognition. It can visualize not only the expression levels of specific molecules in a living system but also specific biological processes, thus providing guidance for early detection and treatment of diseases. As a noninvasive method, imaging agents are one of the foundations of high spatial resolution imaging, and their sensitivity and specificity can be improved by coupling targeting ligands to imaging probes. Among the various targeting ligands (antibodies, aptamers, etc.), targeting peptides are widely used in various modalities of molecular imaging due to their high affinities toward the molecular target and their excellent physicochemical properties. In this review, we summarize the design concepts and methods of targeting peptides in molecular imaging, introduce the combination of targeting peptides and imaging probes in different imaging modalities (e.g., fluorescence imaging, radionuclide imaging), and provide examples of their applications in bioimaging. Finally, the challenges and strategies for clinical translation and practical application of targeting peptide-based imaging reagents are briefly discussed.

## 1 Introduction

Molecular imaging (display of biological processes characterized at the cellular and molecular level in the living body; its key technologies include three aspects: molecular probe technology, systematic measurement technology, and data analysis technology), which provides medical imaging of changes occurring in the human body at the molecular and cellular level, has experienced rapid growth in the two decades since its emergence.^1−3^ Currently, molecular imaging is widely used in early detection of disease,^4^ evaluation of disease treatment,^5^ intraoperative navigation,^6^ and so on. Successful molecular imaging requires not only advanced imaging equipment but also efficient imaging reagents (complexes that bind specifically to a target and produce imaging signals (e.g., light, magnetic, electrical, etc.)).^7^ In general, specific molecular imaging is usually produced by imaging reagents, which are required to have high specificity,^8^ high sensitivity,^9^ high temporal and spatial resolution,^10^ biocompatibility,^11^ and relatively fast blood clearance.^12^ Historically, many of the molecular imaging reagents developed have lacked active targeting capabilities,^13,14^ particularly in tumor imaging, which is thought to reach the lesions based on an enhanced permeation and retention (EPR) effect.^15^ However, such nontargeting imaging molecules may be at risk of more nonspecific binding and resulting side effects.^16,17^ To circumvent these drawbacks and support the unmet need for in vivo clinical molecular imaging, a variety of targeting reagents have been developed, which significantly improve the performance of imaging modalities and profoundly affect the availability of imaging reagents. They usually consist of two components: First, a reporter (fluorescent molecule, radioactive nuclide, etc.) that can provide signals in different imaging modalities, such as computed tomography (CT), positron emission tomography (PET), magnetic resonance imaging (MRI), and fluorescence imaging (FI);^18,19^ second, a targeting ligand that specifically binds to the biomarker of interest and thus transports the reporter to a specific site.^20,21^ The most commonly used targeting ligands today are antibodies that have dominated the field of biomolecule-based affinity regents. However, antibody-based targeting reagents typically produce low-contrast images and low specificity due to the large molecular weight of the antibody (∼150 kDa) and long cycle times^22^ as well as antibody potential to induce an immune-related adverse event (irAE).^23^ Therefore, smaller molecular weight targeting ligands have been widely explored for various applications.^24^ For example, nanobodies (Nbs) lacking light chains are ideal probes for cancer imaging by virtue of their small nanosize (∼13 kDa) and intact variable heavy chain domains (VHHs), and a wide range of imaging reagents based on Nbs has been developed for different tumor markers.^25^ However, the major limitation to the clinical use of Nbs is the risk of nephrotoxicity associated with its high kidney uptake.^26^ Functional nucleic acids (FNAs),^27^ such as aptamers, are another type of recognition element that have the advantage of programmability to visualize a biological process.^28,29^ Besides, there are some small molecules which have been designed and synthesized for binding to biological macromolecules such as proteins and DNA and are now emerging as a recognition element.^30,31^ Several reviews have discussed recent developments in the application of the above targeting ligand molecules in the field of molecular imaging.^32−35^

Notably, targeting peptides (peptides with recognition and binding functions for specific biomarkers), as small-molecule protein fragments, not only have high affinity and specificity toward the relevant targets but also could be chemically modified, so that appropriate peptide-based imaging reagents can be designed for different imaging situations. Compared with mainstream antibodies, targeting peptides are superior in molecular imaging because they have the advantages of having low cost, convenient separation from impurities, and good storage stability.^36,37^ Currently, various peptide-based imaging reagents have been used in targeting disease-related receptors,^38^ biomarkers,^39^ as well as angiogenic processes^40^ and partly are undergoing preclinical and clinical studies.^41,42^ Overall, the general construction principles of targeting peptide-based imaging agents are as follows. The first is to connect the imaging unit to the targeting peptide by using a linker, thereby using the active transport of the targeting peptide to transfer the imaging reagent to the site of interest. However, the targeting peptide and the imaging reagent generally have different physicochemical properties, and their metabolism in vivo is distinctly different, which is avoided as much as possible by selecting an ideal linker. The second is the use of nanocarriers formed from self-assembled targeting peptides to encapsulate the imaging unit, e.g., nanofibers that can be entangled to encapsulate quantum dots and so on. This method is simple but places a high demand on the nature of the peptide assembly. Finally, many imageable small molecules can form noncovalent bonding interactions with targeting peptides, and thus, small-molecule imaging units can be directly involved in the self-assembly of target peptides to form one-piece nanostructures for the construction of imaging reagents. In terms of targeting peptide sources, the conventional strategy is to isolate peptides with specific recognition functions from the physiological environment. For example, the renowned tripeptide RGD (Arg-Gly-Asp) is a shared sequence naturally occurring in extracellular matrix proteins that specifically binds to key structures in cell membrane integrin α_v_β_3_.^43,44^ Its simple structure and ease of modification have made RGD a sought-after building block for imaging reagents.^45,46^ However, with the rapid development in the field of molecular imaging, natural source peptide sequences are far from meeting the application requirements. Recent advances in phage display technology as well as combinatorial chemistry have greatly expanded the sources of targeting peptides, leading to the development of robust strategies for designing peptides as imaging reagents.^47,48^ Indeed, the most appealing aspect of targeting peptides is their ability for self-assembly by rational design.^49^ Combining self-assembled nanostructures with signaling units not only improves the circulating stability of the imaging reagents in blood but also enables multivalent binding to the target.^50^ Moreover, stimulus-responsive peptide assemblies have recently appeared in molecular imaging, which can exploit differences (pH, membrane proteins, etc.) between focal and background tissues to alter the behaviors of peptide assemblies to localize and concentrate them at the target site to improve the signal-to-noise ratio.^51^

In this feature mini-review, we first introduce the concept of a targeting peptide and describe the mechanism of targeting peptide self-assembly (spontaneous formation of ordered nanostructures (e.g., nanofibers, nanotubes, etc.) of peptide molecules in solution by a series of noncovalent bonds (hydrogen bonding, electrostatic interactions, hydrophobic interactions, etc.)); further, we describe the methods of targeted peptide design and screening, mainly focusing on combinatorial chemistry; in addition, we summarize the design of targeting peptide-based imaging reagents in different imaging modalities in recent years. This review may provide a reference for the design and development of medical products based on targeting peptide imaging reagents and allows researchers to review recent advances in the use of peptide-based structures to interface with biology for biomedical imaging applications.

## 2 Targeting Peptide and Self-Assembly

Peptides exist universally in living organisms and are formed by linking 20 naturally occurring amino acids through amide bonds. The history of peptide chemistry can be traced back to around 1900.^52^ With the advent of 9-fluoromethoxycarbonyl (Fmoc) SPPS (solid-phase peptide synthesis), it is now possible to synthesize any peptide for specific sequence and functions, e.g., antitumor peptides, cytokine mimetic peptides, and so on.^53−55^ Notably, with the advent of the era of precision medicine, targeting peptides that can intelligently discriminate and bind to specific targets are coming to the fore among the many recognition molecules.^56^ Compared to common biorecognition molecules (aptamer, DNA, glycan, antibody, etc.), targeting peptides, apart from their shared biocompatibility, high selectivity, and specificity, can form richer sequences due to the diversity of amino acids that make up peptides.^57−59^ Moreover, due to the designability of the targeting peptide, stapled peptides, cyclic peptides, etc., have been developed for molecular recognition scenarios to increase the affinity and stability to the target.^60,61^ However, during targeting peptide recognition, there exists a special class of targets whose binding interface consists of β-strand-rich structures (e.g., PD-L1 and PD-1, etc.).^62^ For such notorious nondruggable targets, it is difficult for small-molecule targeting peptides to bind stably, resulting in poor imaging sensitivity and low spatial resolution.^63^ In fact, the most intriguing property of peptides is their self-assembling properties. With rational molecular design, they can form various secondary structures (α-helix, β-sheet, coiled-coil, β-harpin, etc.) and be further organized into higher order supramolecular morphologies (nanofibers, nanotubes, nanoribbons, etc.).^64,65^ These nanostructures not only enhance the resistance of the targeting peptide to enzymatic degradation in physiological environments but also enable stable binding to the target by means of multivalent binding, which is ideal for imaging targets with large surface structures such as PD-L1.^50^

**Figure 1:**
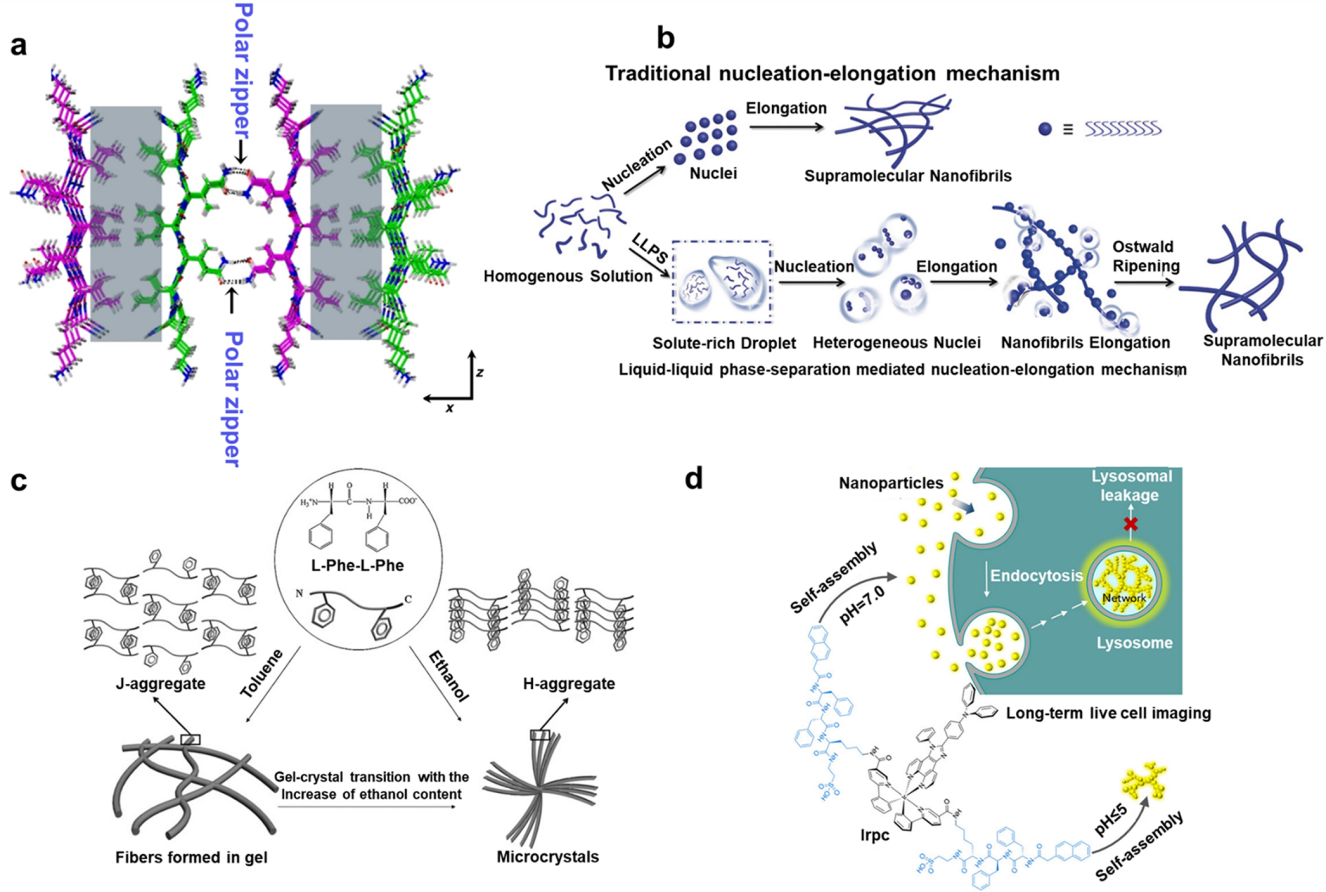
(a) Formation of a polar zipper (black arrows) between neighboring β-sheets. The nonpolar interface is colored gray. Reprinted with permission from ref (68). Copyright 2018 Nature Publishing Group. (b) Schematic illustration of the formation of self-assembling supramolecular nanofibrils by amino acids or short peptides. Reprinted with permission from ref (70). Copyright 2019 Wiley. (c) Schematic illustration of the structural transition induced by varying the ethanol content in the mixed solvents, and the proposed molecular packing in the gel and in the microcrystal. Reprinted with permission from ref (73). Copyright 2010 Wiley. (d) Schematic presentation of the endocytic trafficking of the pH-responsive self-assembly of iridium(III) complex Irpc for long-term lysosome imaging. Reprinted with permission from ref (75). Copyright 2021 Wiley.

Based on the wide variety of amino acids and the diverse properties of the side chains, the peptide self-assembly process involves a variety of noncovalent interactions cooperating with each other. Currently, hydrogen bonding, hydrophobic interactions, π–π stacking, electrostatic interactions, etc., have been well studied. Specifically, hydrogen bonding between peptide backbones directs the formation of secondary structures and drives peptide aggregation in one-dimensional directions, whereas between the side chains of some polar residues it may facilitate the lateral stacking among β-sheets.^66,67^ The hydrogen-bonding network is the most prominent feature of peptide self-assembly. For example, by rationally designing the amphiphilic short peptide Ac-I_3_XGK-NH_2_ (X = N, Q, S), Xu et al.^68^ demonstrated for the first time that hydrogen-bonding interactions between the polar side chains of neighboring β-sheets can form a polar-zipper structure and intertwine many β-sheets with each other to form a flat band (Figure 1a). Besides, hydrophobic interactions exist mainly between some nonpolar residues, and there should be well-defined hydrophobic regions in the peptide that can promote peptide aggregation and thus minimize contact area with water.^69^ During peptide self-assembly, hydrophobic interactions not only drive the aggregation of peptide molecules into “seeds” at the early stage of assembly but also contribute to the further assembly of intact secondary structures. For example, Yan et al.^70^ tracked the dynamic process of the structural evolution of peptide nanofibers by using amphiphilic peptide as a model and found that liquid–liquid phase separation (LLPS) is the key to nucleation of supramolecular molecules, whereas hydrophobic interaction driven formation of disordered droplets is the “compartment” for molecular aggregation and nucleation (Figure 1b). The π–π stacking has been considered as another special hydrophobic interaction because of its directionality as well as the possibility of ordering during self-assembly.^71^ Typical dipeptide FF, whose self-assembly process is dominated by π–π stacking, can be constructed into a variety of functional nanostructures, such as nanotubes, nanowires, and molecular chains, which are widely used in areas such as molecular imaging and biosensing (Figure 1c).^72,73^ Electrostatic interactions existing between the charged amino acids (L, K, R, D, E) are usually distributed on the surface of the nanostructure due to their hydrophilicity, which is essential for the stability of the assembly and recognition with the ligand. Electrostatic interactions are tightly correlated with solution pH; therefore, micro acid tumor microenvironments (TME) or lysosomes can be used to design imaging reagents with stimulus responsiveness for on-demand biomedical applications.^51,74^ For example, for long-term lysosomal imaging, Zhang et al.^75^ synthesized and characterized an aromatic peptide-coupled cyclometalated iridium(III) complex (Irpc). Through endocytosis transport, the acidic environment in the lysosome triggers a self-assembling transformation to form robust stacked nanostructures. In continuous cell culture, biocompatible nanomaterials were delivered to daughter cells for more than 15 generations (Figure 1d). In addition to the various noncovalent bonds mentioned above, metal coordination is also a driving force for the occurrence of peptide self-assembly. Similar to metalloproteins, some residues (E, C, H. etc.) in peptides can drive the formation of high-level structures of peptides by chelating metal ions. For example, Conticello et al.^76^ described an amphiphilic peptide, TZ1H, that forms a trimeric helical coil structure, where histidine residues can be localized in the core hydrophobic center of the trimer providing silver(I) ion binding sites. When it chelates silver(I) ion, it can trigger a conformational transition of the peptide to form nanofibers.

## 3 Targeting Peptide Design and Screening

### 3.1 Peptide Design

**Figure 2:**
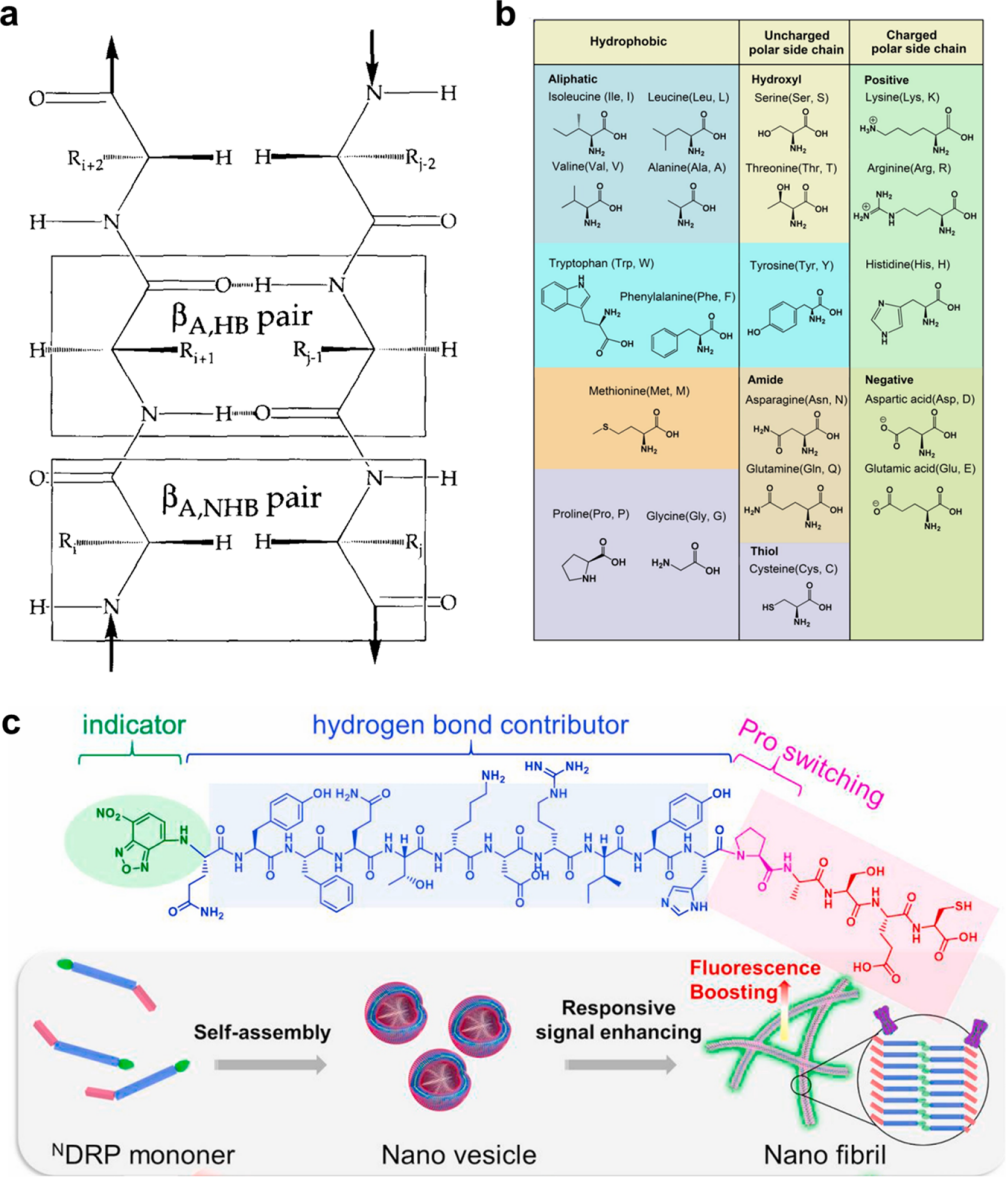
(a) Schematic diagram of hydrogen-bonded sites and non-hydrogen-bonded sites in an antiparallel β-sheet. Reprinted with permission from ref (82). Copyright 1998 Wiley. (b) Twenty natural amino acids and their classification. Reprinted with permission from ref (86). Copyright 2020 Elsevier. (c) P residue-mediated morphological transformation of peptide assemblies in the presence of DDR2 protein. Reprinted with permission from ref (92). Copyright 2021 Elsevier.

Attempts to understand the design rules of peptides and to establish peptide “sequence–structure–property” relationships have been a long-standing pursuit in the field of peptide self-assembly.^77,78^ Early on, attempts were made to use statistical methods to analyze the pattern of amino acid arrangement in the secondary structure of natural proteins (especially β-sheets), and the principle of amino acid pairing (AAP) was proposed.^79,80^ The AAP principle suggests that the backbones of the peptides in a β-sheet remain parallel in orientation and that the paired side chains must be located on the same side of the backbone and extend along the same spatial axis. In addition, when peptide chains are arranged in an antiparallel manner, there are two different types of residue pairs: hydrogen-bonded sites (where the backbone atoms of the residues are hydrogen bonded to each other) and non-hydrogen-bonded sites (where the backbone atoms of the residues are not hydrogen bonded to each other) (Figure 2a). Generally, almost all charged and aromatic residues tend to favor hydrogen-bonded sites, while branched residues tend to favor non-hydrogen-bonded sites.^81,82^ Further, the 26 most probable amino acid pairs in the β-sheet were summarized according to the AAP principle.^79^ The above conclusions can effectively guide the design of peptide molecules, but the AAP principle, which originates from naturally evolved proteins, can no longer meet the demand for diversified peptide properties in existing applications. To further reveal the peptide assembly mechanism and construct new artificial peptide sequences, there have been many reports on de novo design of short amphiphilic peptides.^83−85^ These rule-of-thumb-based design ideas guide how to match amino acids based on the side-chain properties as well as peptide species. Although useful, designing peptides for specific biomedical applications, such as targeting specific proteins to achieve molecular imaging, remains elusive. Essentially, the basis of peptide design is how to select the proper ones from the 20 natural amino acids to build the sequence. It is well known that all amino acids differ by the variation of the side chain attached to the C_α_ atom, so they can be roughly classified according to the side chain into nonpolar amino acids (F, A, L, M, I, W, P, V), polar uncharged amino acids (C, G, Q, N, S, Y, T), and polar charged amino acids (H, K, R, D, E) (Figure 2b).^86^ Besides the above classification, some amino acids have their own unique role in the self-assembly process. For example, G, as the simplest amino acid with a side chain of only hydrogen atoms and no apparent hydrophilicity or hydrophobicity, is commonly used as a linker group between different functional units. Moreover, as the only nonchiral amino acid, it has recently been shown to have an unexpected role in regulating supramolecular chirality due to its preference for a single β-strand conformation.^87^ Phosphorylation of the side chain of the Y residue and its dephosphorylation under alkaline phosphatase (ALP) have been widely used in the enzyme-induced self-assembly (EISA) of peptides.^88,89^ This strategy enables intelligent transformation of peptide nanostructures in the tumor microenvironment and can fully exploit the advantages of peptide assemblies in tumor therapy and imaging.^90,91^ The P residue is a terminator of the α-helix due to its unique molecular structure (the side chain is directly connected to the backbone). In β-sheets, it is also unable to form a hydrogen bond with neighboring β-strands, leading to reduced stability of the assemblies. Exploiting this property of P residues allows the design of peptide assemblies with receptor-induced deformation, which can fully satisfy the needs in different physiological environments. For example, we have previously inserted P residues specifically into discoidin domain receptor 2 (DDR2)-targeting peptides, so that they could self-assemble into nanocapsules and load the drug Chlorin e6 (Ce6) under a physiological environment. Upon reaching the tumor tissue, P residues can disrupt the nanocapsules and transform them into nanofibers when induced by DDR2, achieving damage to tumor mitochondria (Figure 2c). This strategy is fully compatible with the advantages of a high drug loading capacity of nanocapsules as well as high stability and multivalent targeting of nanofibers.^92^

**Figure 3:**
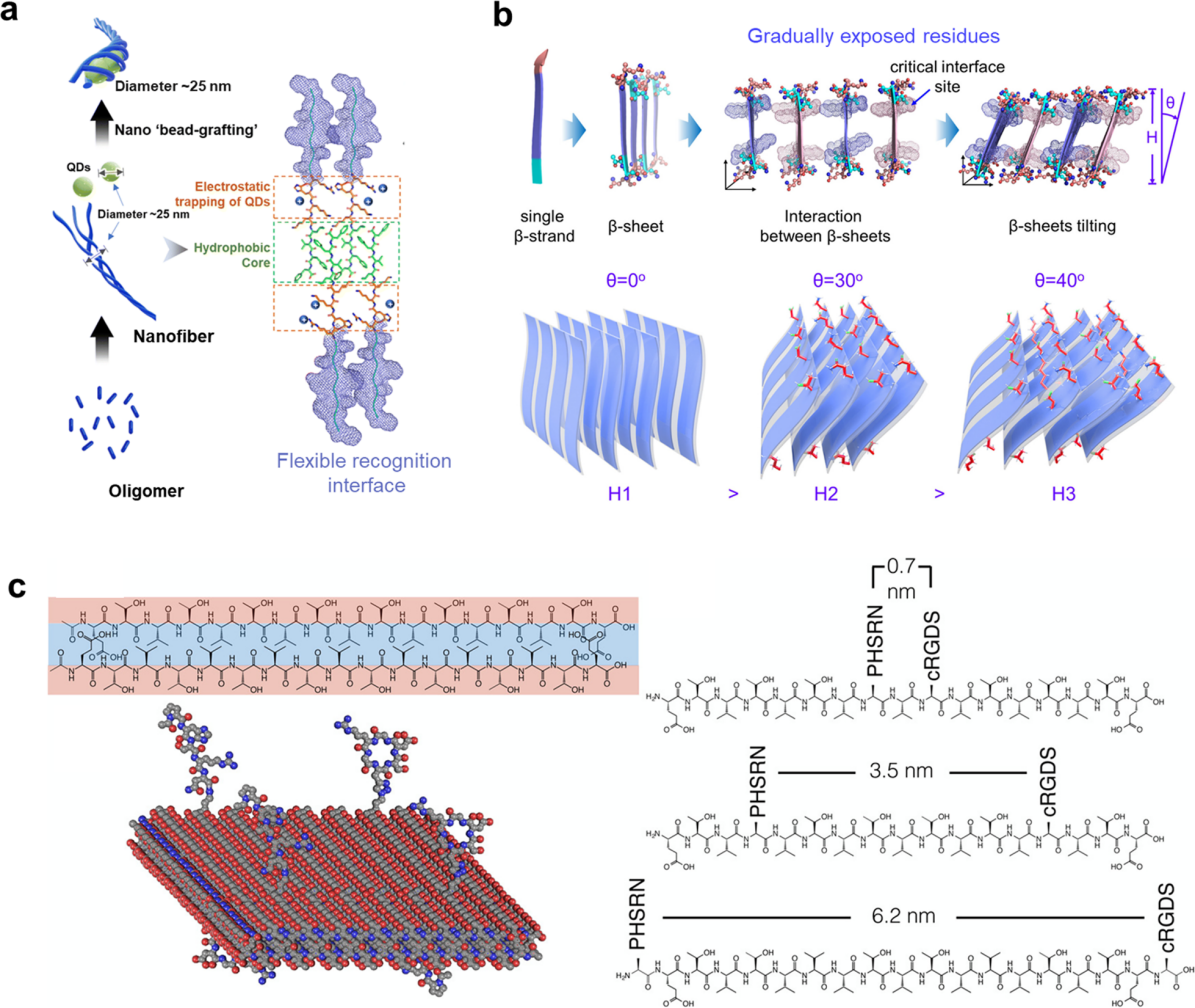
(a) CD47 targeting peptide RT encapsulates Ag_2_S quantum dots in a self-assembly pathway to form a “bead-grafting” nanostructure. Reprinted with permission from ref (99). Copyright 2022 Royal Society of Chemistry. (b) “Collapsed peptide dominos” diagram with different tilted angles (θ) for controllable β-sheet assembly (θ = 0°, β-sheets are perpendicular to the surface of the assembly, and no residues are displayed; at θ = 30°, four residues are displayed; at θ = 40°, six residues are displayed.). Reprinted with permission from ref (105). Copyright 2022 Wiley. (c) Alternating hydrophobic and hydrophilic peptide sequences to form a bilayer structure and control of the different distances between the control PHSRN and the cyclic RGDS. Reprinted with permission from ref (106). Copyright 2016 American Chemical Society.

In addition to the selection of appropriate amino acids, the identification of the appropriate peptide type according to the specific application is also crucial for designing peptide self-assembly systems. Common types of peptides are as follows: ionic complementary peptides consist of alternately arranged charged amino acids and hydrophobic amino acids, and their rich charged nature confers them with unique biological activities, making them well suited for applications in stimuli-responsive molecular imaging and other applications.^93−95^ However, this class of peptides must be designed to ensure ionic complementarity, which greatly limits sequence diversity. Moreover, the abundance of charges raises safety and stability concerns for in vivo applications.^96^ Surfactant-like peptides (SLPs) are one of the most studied classes of peptides. A typical SLP consists of two parts: a hydrophobic tail composed of several consecutive hydrophobic amino acids and a hydrophilic head composed of one or two hydrophilic amino acids.^97^ During self-assembly, hydrophobic-tail-dependent interactions enable rapid aggregation of peptides and formation of nanofibers under a series of noncovalent bonds such as hydrogen bonding. Notably, the assemblies formed by SLPs can display hydrophilic sequences on the surface of the assemblies, which can be exploited to display functional units (target recognition, epitopes, etc.) specifically on the surface of the assemblies to achieve tumor specific imaging.^98^ We previously designed an SLPs peptide RT with high affinity for the immune checkpoint cluster of differentiation 47 (CD47), which can display recognition units on the surface of nanofibers to specifically recognize tumor-derived rather than red blood cell-derived CD47. Furthermore, its unique self-assembling pathway can encapsulate Ag_2_S quantum dots to form a nano-“ bead-grafting” structure. We demonstrated that this unique structure specifically indicates the site of the tumor with less effect on normal tissue (Figure 3a).^99^ Bola-like peptides (special amphiphilic peptides with hydrophilic residues at both ends and hydrophobic residues in the middle) have a special symmetrical structure.^100^ This class of peptides tends to fully stretch the peptide chain during assembly, with a tendency to form nanotubes as well as nanoribbons for applications such as gene vectors.^101^ Notably, the types of peptides described above all self-assemble based on the β-sheet. However, in the β-sheet structure, the peptides share the common “cross-β” feature. The current structure resolution of the cross-β assemblies reveals that the β-sheets are arranged vertically, so that adjacent sheets are fully complementary and the side chains can engage with each other.^102,103^ This makes multiple noncovalent bonding interactions in the assemblies highly coupled,^104^ causing severe interference in molecular imaging and therapy based on the binding of targeting peptides to the ligand. To attempt to solve the above problem, we obtained bola-like peptides using de novo design and combinatorial chemical screening method. By regulating the solvent-accessible surface area of the peptide chain, a series of assemblies with different tilt angles and active sites of the β-sheet was obtained, resembling collapsed dominos (Figure 3b). We demonstrated at the molecular, cellular, and in vivo levels that such structure can be adequately adapted to target PD-L1. Therefore, it has potential application in the precision diagnosis and treatment of PD-L1 positive tumors.^105^ In addition, reasonable control of the spatial distribution of active units on the peptide assemblies is also an effective way to solve the above problems. Stevens et al.^106^ developed a strategy for spatial control of ligands on peptide assemblies. They attached the fibronectin-derived peptides RGDS and PHSRN to variably spaced side chains on a β-sheet peptide scaffold. Supramolecular fibrils were prepared by coassembly of the unmodified assembly motif with a functionalized variant in which the ligands were arranged in 0.7, 3.5, and 6.2 nm spacings as determined for ideal α_5_β_1_ integrin activation; HUVEC cell cultured results showed that the nanofibers with the correct ligand spacing were found to bind cells more effectively (Figure 3c).

**Figure 4:**
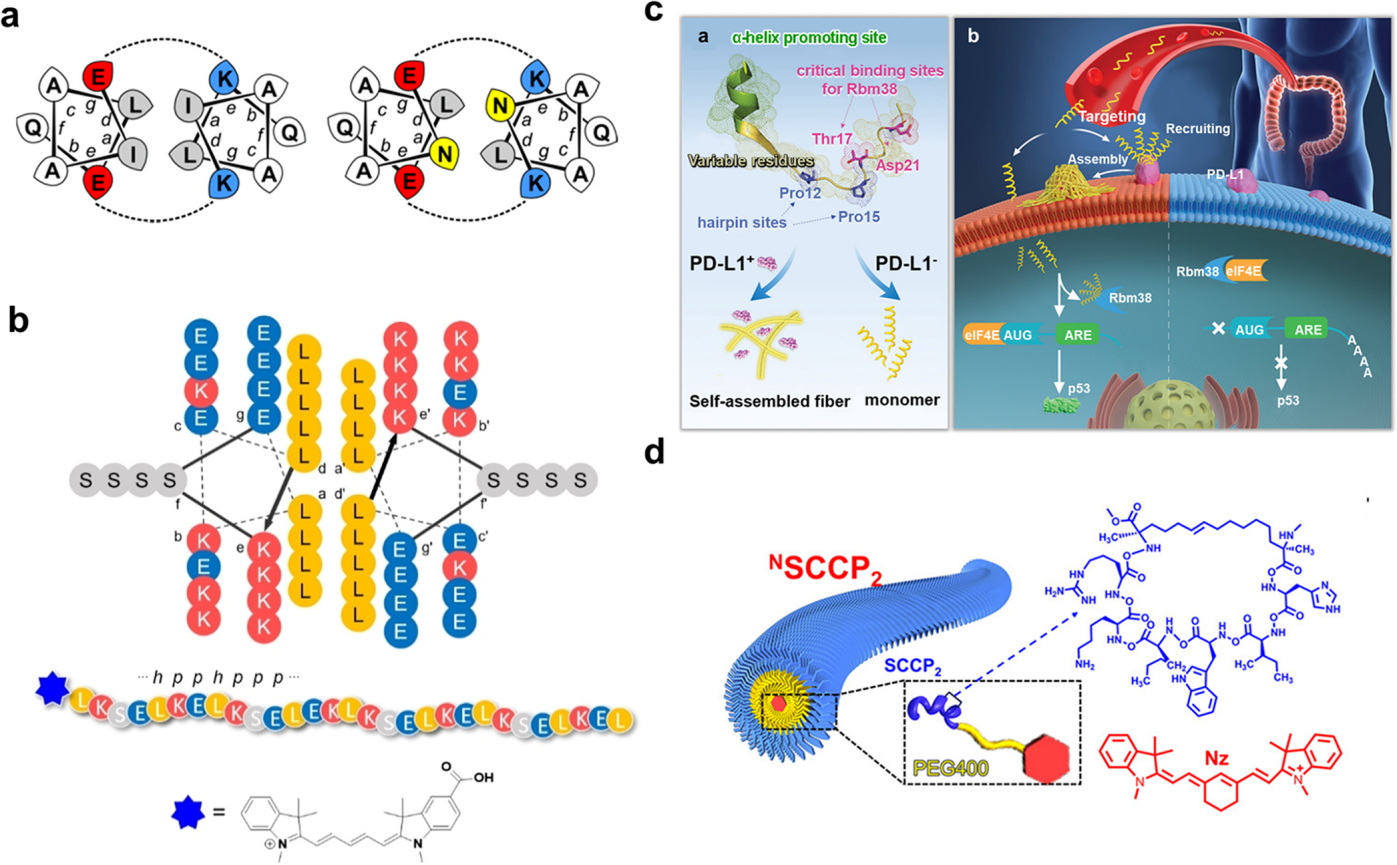
(a) Helical wheel representation of the primary sequence of STAP1 and STAP2. Reprinted with permission from ref (110). Copyright 2013 American Chemical Society. (b) Linear representation of STAP1 showing the *N*-terminal Cy5 dye indicated by a blue star. Reprinted with permission from ref (113). Copyright 2022 Wiley. (c) The “all-in-one” peptide TAP is converted to an α-helical structure in TME and assembles into dense nanofibers recruited by PD-L1, thereby blocking the PD-1/PD-L1 axis. Upon entry into tumor cells, it blocks the formation of the Rbm38-eIF4E complex, thereby upregulating p53. Reprinted with permission from ref (114). Copyright 2023 Wiley. (d) Molecular structure of CD133-targeted stapled peptide. Reprinted with permission from ref (117). Copyright 2021 American Chemical Society.

In addition to the β-sheet design mentioned above, α-helical is the most common secondary structure in natural proteins and plays a key role in protein–protein interactions (PPIs) as recognition motifs, with 62% PPIs mediated by α-helical structures at the interface.^107^ It follows that designing peptides that mimic the natural α-helical structure may be more popular for molecular recognition applications. From a self-assembly perspective, different noncovalent interactions play different roles in α-helical self-assembly, with backbone hydrogen bonding being responsible for the formation of the helical structure of the peptide chain while the side chains are further driven to form the ordered structure, and a large number of side-chain motifs can be displayed on the surface of the nanostructures for binding to the target.^108^ Woolfson et al. have done extensive research and summarized universal molecular design rules in self-assembly based on coiled-coil structures.^109,110^ For example, coiled-coil sequences have a heptad pattern of hydrophobic (h) and polar (p) residues, i.e., hpphppp, which is usually represented as abcdefg. This residue arrangement guides the folding of amphipathic helices, which associate through their hydrophobic (a/d) faces (Figure 4a). The helices are intertwined, in which interhelical side chains pack in a characteristic “knob-in-hole” manner.^111,112^ Following this strategy, Koksch et al.^113^ de novo designed the pH-sensitive coiled-coil sequence and further coupled the cyanine dye Cy5 at its N-terminal end. This molecule demonstrated pH-responsive assembly behaviors and exhibited spectral features of H-aggregates, which could be further applied in tumor imaging (Figure 4b).

In addition to coiled-coil molecules, there have been other designs for α-helical assembly molecules. For example, to achieve simultaneous blockade of the PD-1/PD-L1 axis and activation of p53, we used RNA-binding motif protein 38 (Rbm38) and PD-L1 as targets and the α-helical backbone as a template to design a peptide library. By using the “3D-molecular-evolution”, we screened a totipotent “all-in-one” peptide (TAP). In the TME, TAP can transform into α-helical structures and assemble into dense nanofibers under the recruitment of PD-L1, thus blocking the PD-1/PD-L1 axis. After entering the tumor cells, it blocks the formation of the Rbm38-eIF4E complex, thus upregulating p53 (Figure 4c).^114^ It should be noted that the α-helical entropy value is low and the hydrogen-bonding network is blocked inside the peptide, leading to its poor stability. Currently, various strategies have been used to try to improve the above problems. An example is the stapled peptide strategy, which greatly improves not only the rigidity of the peptide helical structure but also the affinity to the ligand.^115,116^ In our previous study, we proposed a peptide-directed evolution strategy and succeeded in obtaining an α-helical peptide capable of targeting the glioma marker Prominin-1 (CD133), which was further modified using an all-hydrocarbon peptide stapling strategy as well as a NIR-II fluorescent probe (Nz) to confer its high affinity and signaling unit. Its ability to self-assemble into nanofibers relying on carbon chains and aromatic Nz units enables effective in situ imaging of gliomas (Figure 4d).^117^ In addition to the stapled peptide strategy, there are also many reports on the stabilization of α-helix self-assembly by means of coordination between metal ions and the peptide backbone.^104,118,119^

### 3.2 Targeting Peptide Screening

Isolation from organisms was the earliest method of obtaining targeting peptides, but it is currently unable to fundamentally meet the requirements for application.^120^ The development of various peptide screening techniques has effectively compensated for the lack of targeting peptides. Phage display technology utilizes bacteriophage to display targeting peptides on their surface through insertion of the gene encoding the corresponding peptides into the phage genome. The library’s diversity (up to 10^11^) and its rapid and efficient features make it a powerful technique for screening target-specific peptides.^121,122^ More discussion about this section has been given in detail in other reviews and is therefore not the focus of our discussion.^123,124^

One-bead-one-compound (OBOC) libraries based on solid-phase peptide synthesis are another method for peptide screening. The “split-and-mix” is a common peptide library synthesis strategy.^125^ The main advantage of the OBOC peptide library is its high compatibility with a wide range of building blocks (e.g., unnatural amino acids, fluorescent molecules, etc.).^126^ Since OBOC can obtain a large number of peptides in a short period of time, randomly large and diverse peptide libraries are a common strategy for constructing peptide libraries. Resolving the crystal structure between the target and the ligand and using computational modeling contributes to the generation of peptide libraries.^127^ This approach may rationalize the discovery of novel targeting peptides in the future.

**Figure 5:**
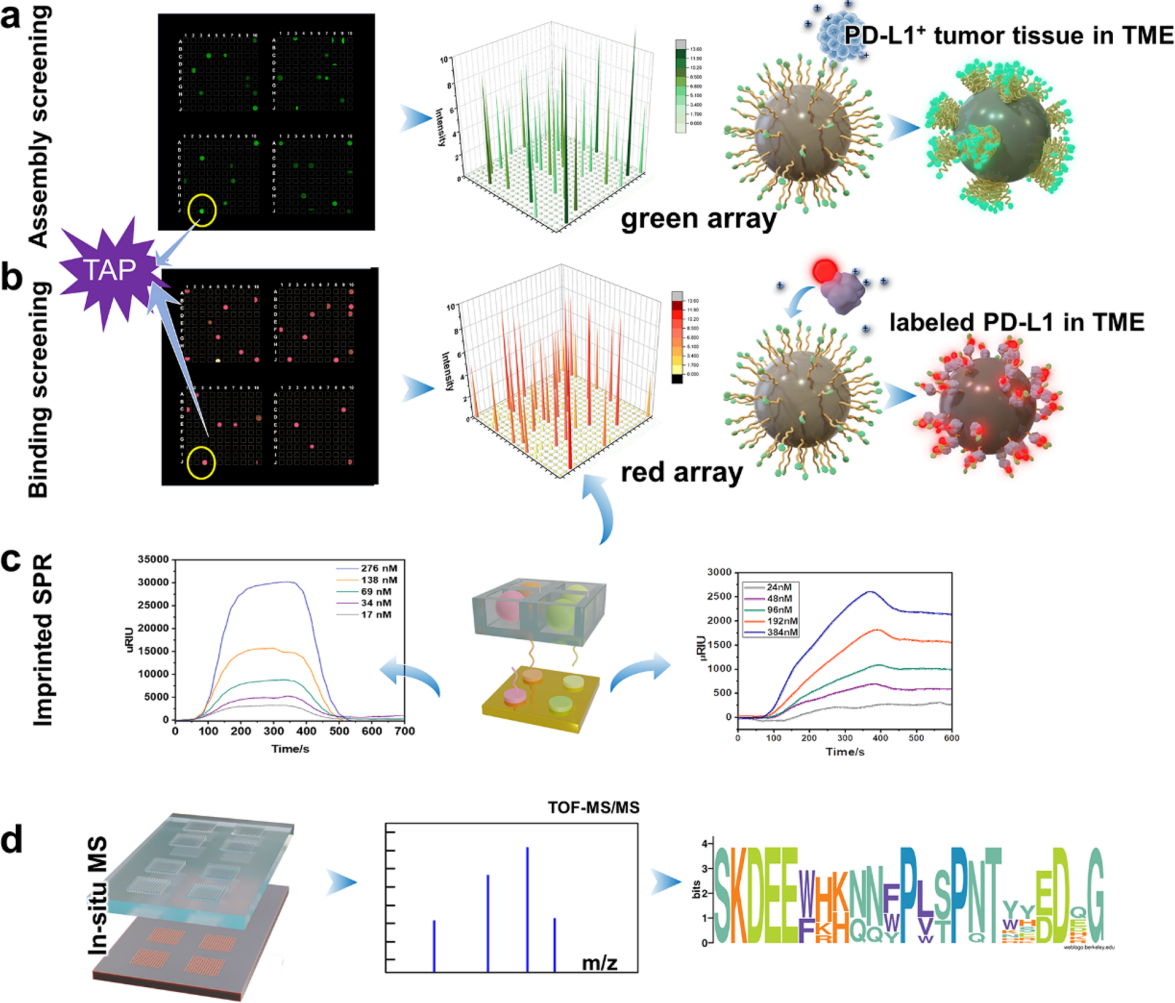
“3D-molecular-evolution” and “screening–imprinting” strategy. (a) First dimension of evolution on a solid-phase support: self-assembled peptides located in a PD-L1^+^ tissue environment by fluorescence “turn-on”. (b) Second dimension of evolution on a solid-phase support: PD-L1 targeting peptide sequence screening through a microarray detection in TME. (c) “Imprinted” microarray for the in situ SPR detection as well as the SPRi results between TAP and PD-L1. (d) In situ MALDI-TOF-MS sequencing and the alignment of all the TAPs. Reprinted with permission from ref (114). Copyright 2023 Wiley.

In the screening of OBOC peptide libraries, traditional methods usually rely on visual observation of positive beads under a fluorescent microscope, which is a labor-intensive process. Automated sorting instruments have been developed to improve the efficiency and accuracy of positive bead sorting, but they are limited to fluorescence-based peptide bead sorting and are expensive.^128,129^ Magnetic labeling combined with microfluidic screening provides a simple and reliable strategy for screening positive beads.^130^ When combined with tandem mass spectrometry, it provides a convenient method for the resolution of positive peptide sequences. However, the screening and identification of target peptides involves the collection and cleavage of peptide beads, peptide sequencing, resynthesis and purification, and affinity identification. The tedious steps are exhausting. To accelerate the screening efficiency of desirable peptides, OBOC peptide libraries have been combined with a variety of devices (e.g., microfluidics, etc.) and significant progress has been made. However, most of the existing targeting peptides were screened in vitro using recombinant proteins as targets, but the real physiological environment is full of interferences such as enzymes, and the peptides face the problem of insufficient stability and specificity. To address the above issues, we tried to obtained a totipotent “all-in-one” peptide TAP from the OBOC library by developed “3D-molecular-evolution” combined with a “screening-imprinting” strategy using tumor tissues as an environment, which possesses the abilities of self-assembling, blocking the PD-1/PD-L1 axis, inhibiting Rbm38-eIF4E complex formation, and activating p53 (Figure 5a–f).^114^ This platform allows access to targeting peptides with multiple properties (self-assembly, stability, specificity, targeting, etc.) in a short period of time, providing a new molecular screening strategy for imaging and therapeutic reagents.

Other established methods that have been developed to screen targeting peptides include the spatially addressable parallel library method, synthetic library methods requiring deconvolution, the affinity selection method, etc.

## 4 Construction of Targeting Peptide-Based Imaging Reagents

Once the target-specific peptides have been screened, they can be labeled with imaging units of different imaging modalities to provide imaging signals. Common labeling units include radioisotopes, near-infrared fluorophores, etc. Depending on the labeling unit, different imaging reagents can be used for different modalities of imaging, such as magnetic resonance imaging and fluorescence imaging, among others. However, each imaging modality has its own advantages and disadvantages; it is necessary to choose the best imaging modality depending on the type of biological information one wishes to collect when designing imaging reagents.

### 4.1 Radionuclide Imaging

**Figure 6:**
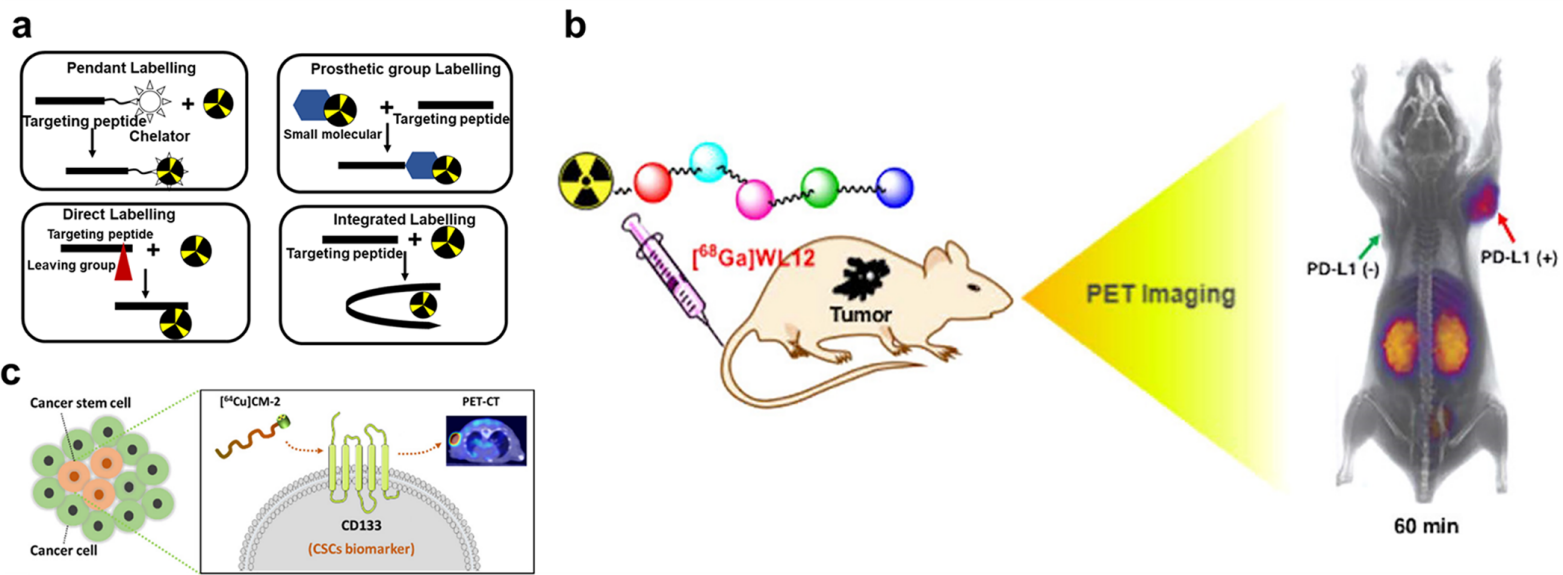
(a) Various methods for introducing a radioisotope into a peptide sequence. (b) [^68^Ga] WL12 can be used with PET to distinguish PD-L1 positive tumors and quantify PD-L1 expression in tumors. Reprinted with permission from ref (138). Copyright 2018 American Chemical Society. (c) [^64^Cu]CM-2 was designed as a PET radiotracer for CD133 to detect the cancer stem cell populations in tumors. Reprinted with permission from ref (140). Copyright 2022 American Chemical Society.

While multiple imaging modalities are rapidly evolving, radionuclide imaging remains the preferred choice for clinical patients. This is mainly due to the high sensitivity of the tissue imaging that can be performed with the minimum amount of radioactive imaging agents.^131^ Single-photon emission computed tomography (SPECT) and positron emission tomography (PET) make up radionuclide imaging. Among them, SPECT has the advantage of gamma imaging, and the main radioisotopes used in SPECT are technetium-99m [^99m^Tc], iodine-123 [^123^I], and indium-111 [^111^In], etc. PET is another very sensitive molecular imaging modality, where imaging agents are usually radiolabeled with a proton-rich radionuclide that emits a positive charge (β^+^) during decay.^132^ The physical principles of PET allow more choices in radionuclides for imaging tracers. For example, carbon-11 [^11^C], nitrogen-13 [^13^N], fluorine-18 [^18^F], gallium-68 [^68^Ga], copper-64 [^64^Cu], and zirconium-89 [^89^Zr], and so on, can be used.^133^ Moreover, to introduce the above radionuclide into the targeting peptide, a ligand must first be chelated to it. There are four main strategies: pendant labeling, integrated labeling, prosthetic group incorporation, or direct labeling (Figure 6a). It is common that a portion of the radionuclide can be attached to the peptide by direct chelating via 1,4,7,10-tetraazacyclodecane-1,4,7,10-tetraacetic acid (DOTA), 2,2′,2″-(1,4,7-triazacyclononane-1,4,7-triyl) triacetic acid (NOTA), and so on.^134,135^

Currently, a variety of targeting peptides have been used for PET/SPECT imaging, for example, the RGD sequence and its analogues (cyclic RGD analogues, RGD multimers, etc.) that specifically bind to tumor cell surface integrins (α_v_β_3_).^136^ [^18^F]FRGD_2_ and [^18^F]FPPRGD_2_ were approved by clinical investigation.^137^ Nimmagadda et al.^138^ prepared the imaging agent [^68^Ga]WL12 by combining the targeted peptide W12 (IC_50_ ≈ 23 nM), which has a high affinity for PD-L1, with ^68^Ga. The imaging agent can be used to noninvasively quantify PD-L1 expression in tumors by PET (Figure 6b). The octreotide analogue is another peptide widely used in radionuclide imaging with high affinity for somatostatin receptors (SSTrs).^139^ Beyond that, our group developed a clinically relevant, stable, and targeting peptide-based positron emission tomography (PET) tracer, [^64^Cu]CM-2, for mapping CD133 protein in several kinds of cancers. This tracer exhibited specific binding to CD133-positive cancer stem cells in multiple preclinical tumor models and showed durable tumor retention and fast renal clearance (Figure 6c).^140^

### 4.2 Fluorescence Imaging

Fluorescence imaging (FL) is a noninvasive modality with high sensitivity, no radiation, harmlessness, as well as simplicity of operation.^141^ However, autofluorescence interference from biological tissues and the low depth of light penetration limit the clinical application of FL. Near-infrared (NIR, 650–1700 nm) with its low autofluorescence, deep tissue penetration, and small light scattering characteristics provides high resolution as well as signal-to-background scaling and has been demonstrated to be an effective real-time imaging method. Notably, the NIR-II window (950–1700 nm) shows lower photon scattering in neighboring tissues with higher spatial resolution compared to the first NIR-I region (650–950 nm).^142−145^ These advantages make NIR-IIa (1300–1400 nm)/IIb (1500–1700 nm) imaging satisfactory for high-resolution and deep-tissue imaging.

A variety of highly sensitive fluorophores having different characteristics, from small molecules to nanoparticles, have been developed to meet imaging needs in a variety of environments. Among them, small-molecule fluorophores have a well-defined molecular structure, high purity, and reproducibility. Common fluorophores in the NIR-I region including polymethine cyanines, phthalocyanines, porphyrin derivatives, squaraine derivatives, and so on.^146^ The most representative NIR-I fluorophore is indocyanine green (ICG), which is the only NIR fluorophore approved by the U.S. Food and Drug Administration (FDA) for clinical NIR imaging. However, its quantum yield in aqueous solution is only 0.01. And, it suffers from short blood circulation, serum instability, high liver uptake, and being nontargeting.^147^ Besides, various nanoparticles with NIR prosperity have been synthesized for imaging, including quantum dots (QDs), rare-earth nanoparticles, gold nanoparticles, and so on.^148−150^ Compared to small molecules, nanoparticles possess higher stability, longer blood circulation times, and more. However, they also have the limitations of being complex to design, being difficult to produce on a large scale, and having unknown long-term toxicity in vivo.^151^

**Figure 7:**
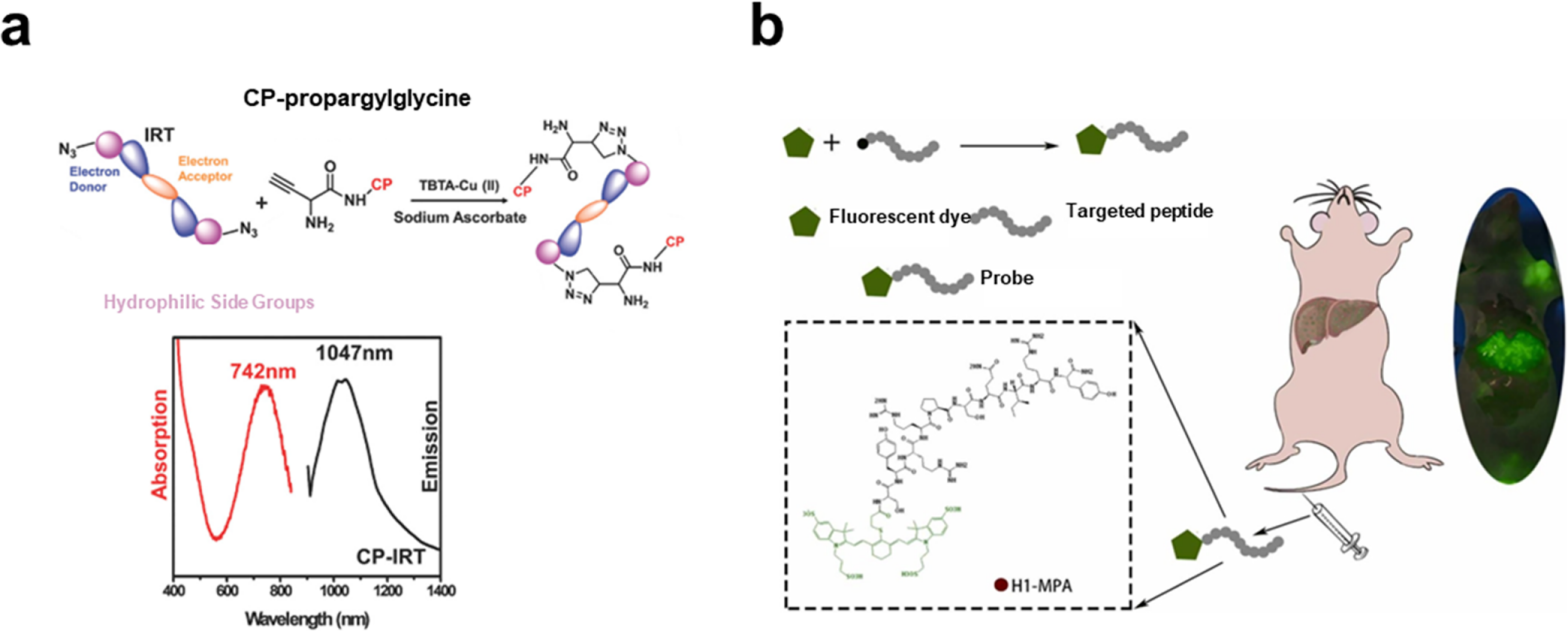
(a) The structures and spectrum of CD133 targeting NIR-II dye–peptide probe CP-IRT. Reprinted with permission from ref (152). Copyright 2018 Wiley. (b) Structure of H1-MPA, a near-infrared probe for targeting EGFR peptides. Reprinted with permission from ref (153). Copyright 2023 Elsevier.

By combining specific targeting peptides with the above fluorophores, imaging reagents can be more precisely delivered to the interested focus compared to the enhanced permeability and retention (EPR) effect. Generally, small-molecule fluorophores can be directly linked to the target peptide through covalent bonds. We developed a peptide CP specific for the tumor stem cell marker CD133 (*K*_D_ ≈ 7 × 10^–9^ M) and prepared CP-IRT based on a click-chemical reaction linking it to a near-infrared organic fluorophore. CP-IRT has an ∼300 nm Stokes shift and a high tumor-to-normal tissue signal ratio (T/NT > 8). Importantly, its excretion in the kidney is rapid (∼87% excretion within 6 h), and we performed the first noninvasive fluorescence imaging of the urinary tract from intact mouse tissue (Figure 7a).^152^ Gu et al.^153^ synthesized a targeting peptide H1 with high affinity for epidermal growth factor receptor (EGFR) and further coupled it with a near-infrared fluorescent molecule, MPA, to synthesize a probe, H1-MPA, which has a high tumor uptake rate and signal-to-noise ratio and can be used to locate tumor boundaries (Figure 7b). In addition, we designed and synthesized a stapled peptide NSCCP2 with high affinity for the glioma marker CD133, which linked to the fluorophore Nz could penetrate the blood–brain barrier (BBB) for in situ imaging of gliomas.^117^ For the nanoparticles class of fluorophores, targeted delivery can also be achieved by modifying targeting peptides or antibodies on their surfaces, but this strategy requires complex synthetic synthesis schemes, which are often difficult to predict and control.^154,155^ In this context, we attempted to use noncovalent bonds for efficient delivery of nanoparticle fluorophores. We designed a surfactant-like peptide library and successfully screened the peptide RT (*K*_D_ ≈ 3.18 × 10^–7^ M) with high affinity for the immune checkpoint CD47. This peptide was able to self-assemble into nanofibers and could efficiently capture Ag_2_S quantum dots in its self-assembly pathway relying on noncovalent bonding interactions. In vivo imaging results demonstrated that this imaging reagent was efficiently enriched in tumor tissue and remained stable for a long period of time.^99^

### 4.3 Magnetic Resonance Imaging

**Figure 8:**
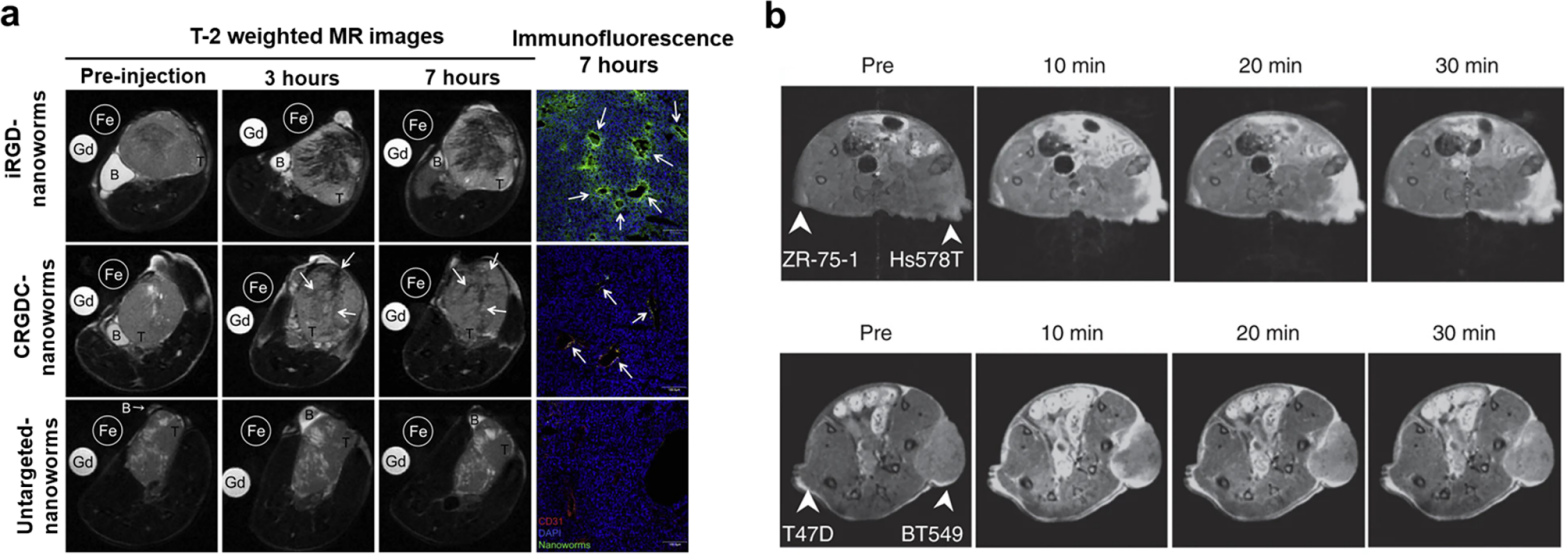
(a) Tumor imaging with iRGD-coated iron oxide nanoworms. Reprinted with permission from ref (163). Copyright 2009 Elsevier. (b) Molecular MRI with ZD2-Gd_3_N@C80 of other breast tumors in mice. Reprinted with permission from ref (164). Copyright 2017 Nature Publishing Group.

Magnetic resonance imaging (MRI) has been widely used in clinical diagnostics.^156^ MRI is a noninvasive technique with the advantages of having extremely high spatial resolution and the absence of radiation exposure.^157−160^ This feature allows early detection of even tumors as small as a few hundred cells and hundreds of micrometers in size.^161^ However, MRI has low sensitivity and usually requires a contrast agent to increase its sensitivity. Generally, MRI contrast agents are broadly classified into longitudinal relaxation contrast agents (T1 preparation) and transverse relaxation contrast agents (T2 preparation). T1 reagents have high signal intensity and relatively bright T1-weighted images that can be used for anatomy. As a comparison, the T2-weighted image of the T2 reagent is darker with lower signal intensity, which is favorable for lesion observation. Paramagnetic materials, including superparamagnetic iron oxide nanoparticles, among others, are often used as contrast agents to increase the T1 and T2 relaxation rates of water protons in tissues of interest and to produce contrast enhancement for diagnostic imaging.^162^ Sugahara et al.^163^ constructed a targeting MRI contrast agents by integrating the cyclic peptide IRGD (CRGDK/RGPDC) onto magnetic nanoworms. After intravenous injection, T2-weighted MRI signals of integrin and neuropilin-expressing tumors were significantly reduced, and local fluorescence of tumors was enhanced (Figure 8a). Besides, Lu et al.^164^ synthesis of a highly relaxation-targeted contrast agent ZD2-Gd3N@C80 for sensitive molecular magnetic resonance imaging of breast cancer by coupling the targeting peptide ZD2 to hydroxylated Gd3N@C80. This contrast agent has superior T1 relaxation, approximately 20 times that of conventional Gd-based MRI contrast agents. MRI with a reduced dose of contrast agent produces strong signal enhancement in multiple aggressive triple-negative breast cancer (TNBC) tumors, whereas the effect is less pronounced in low-risk breast tumors (Figure 8b).

### 4.4 Photoacoustic Imaging

**Figure 9:**
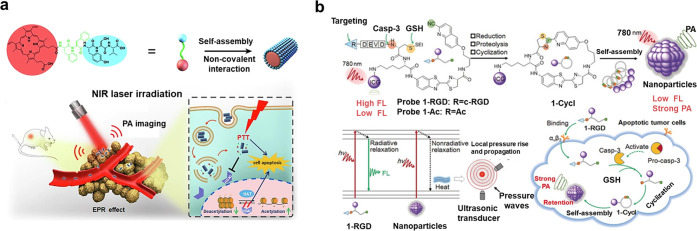
(a) Schematic representation of the molecular structure of PpIX-FFYSV and the self-assembly of the monomer into nanorod-like nanoaggregates (PpIX NAs) for in vivo tumor photoacoustic imaging. Reprinted with permission from ref (166). Copyright 2021 Elsevier. (b) Chemical structures of 1-RGD, and schematic of the mechanism of 1-RGD for PA imaging of caspase-3 activity in apoptotic tumor cells. Reprinted with permission from ref (167). Copyright 2019 Wiley.

Photoacoustic imaging (PAI) is based on the principle of thermal expansion of an object due to absorption of light. As an emerging imaging technique, PAI has great potential to enhance ultrasound and enrich optical contrast. The advantages of PAI are the potential for high spatial/temporal resolution and the absence of ionizing radiation, etc.^165^ Currently, a number of PAI probes based on NIR properties have been developed, such as ICG, porphyrin, etc. Liu et al.^166^ constructed an all-in-one self-delivery nano system by coupling the photosensitizer porphyrin (PpIX) with functional peptide units and further by self-assembly. After intravenous injection, the in vivo tumor inhibition rate of the combined chemotherapy–photothermal therapy was up to 70% in a single injection guided by PAI, which allowed a significant reduction of the chemotherapy dose (Figure 9a). Ye et al.^167^ reported an activatable caspase-3 PA imaging probe (1-RGD) based on a biocompatible response-mediated self-assembly strategy by integration with a tumor-targeting ligand (i.e., c-RGD) and a PA imaging dye. 1-RGD can be effectively activated by active caspase-3 in apoptotic tumor tissues, resulting in a strong PA signal to detect caspase-3 activity. Using reconstructed 3D PA images, 1-RGD can accurately report the location of caspase-3 activation in tumor tissues and is expected to be used for early monitoring of apoptotic tumor response to chemotherapy. (Figure 9b), which may be useful for noninvasive cancer imaging and monitoring.

### 4.5 Multimodality Imaging

**Figure 10:**
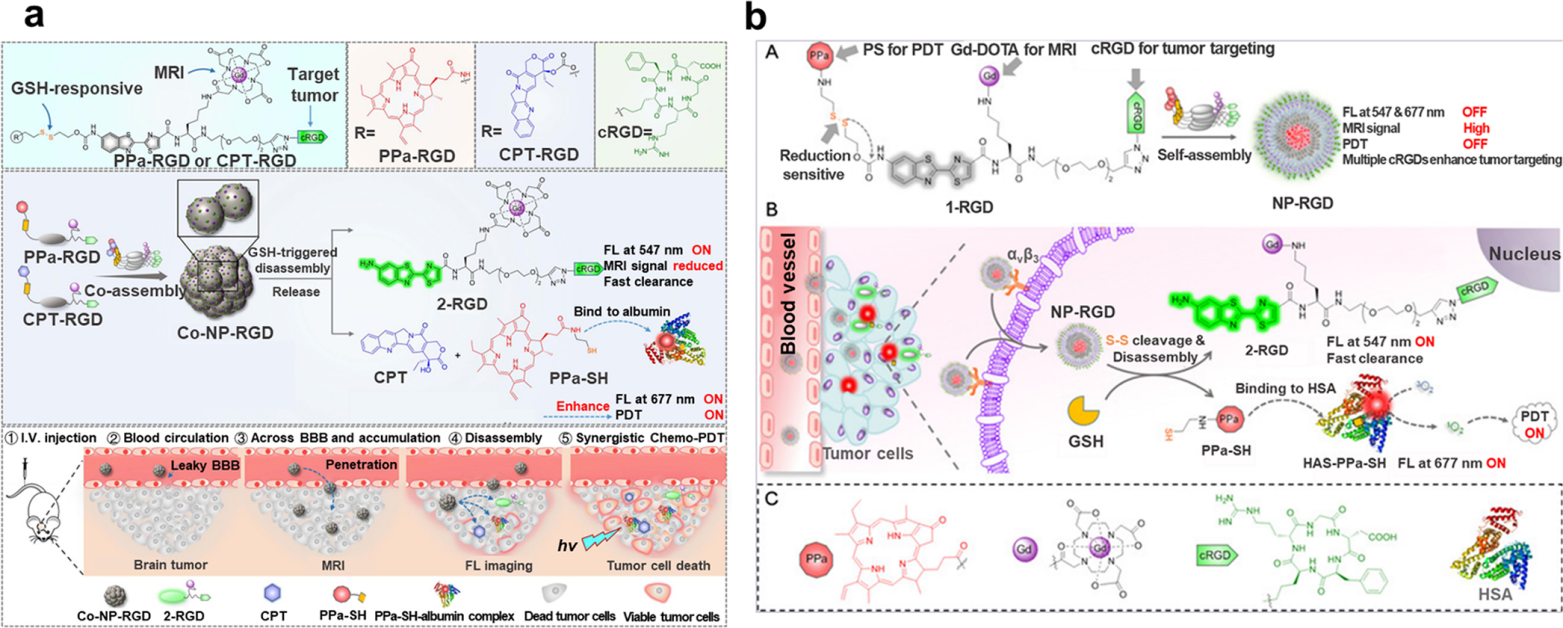
(a) Design principle of the coassembled nanoparticle CoNP-RGD1/1, and schematic diagram of its process of generating magnetic resonance and near-infrared FL signals. Reprinted with permission from ref (172). Copyright 2022 American Chemical Society. (b) Construction of a NIR/MRI dual-modality imaging nanoprobe, NP-RGD, and principles of imaging and generating PDT effects in tumor cells. Reprinted with permission from ref (168). Copyright 2020 Wiley.

The various single-mode imaging modalities mentioned above have been deployed in a variety of biomedical applications and can provide references for disease diagnosis. However, there are inherent shortcomings of the various imaging modalities, such as FL imaging has inherent penetration depth limitations, PET imaging has low spatial resolution, while PA imaging is difficult to perform whole-body MRI and obtain quantitative images due to its low sensitivity. Multimodality imaging, a combination of imaging modalities, can overcome the limitations of the independent techniques, potentially producing comprehensive anatomical and/or molecular information for improved in vivo imaging.^168−170^ In the past few years, there has been an explosion of research on the development of multimodal contrast agents.^171^ Ye et al.^172^ developed a glioma-targeting and redox-responsive coassembled nanoparticle (CoNP-RGD1/1) using a coassembly and redox-mediated disassembly strategy. Co-NP-RGD 1/1 is able to cross the blood–brain barrier and can aggregate in αvβ3-positive gliomas in the presence of the targeting peptide RGD, which produces bright MR contrast (>53% SE) and enhanced NIR FL signals in in situ U87 MG tumors, allowing for noninvasive and precise localization of tumor sites in mouse brains. Furthermore, Co-NP-RGD 1/1 combined with chemo-PDT inhibited the progression of subcutaneous and in situ U87 MG gliomas and significantly prolonged the median survival of mice as demonstrated by FL and MR dual-modality imaging results (Figure 10a). In addition, through self-assembly and redox-driven decomposition strategies, this group also reported a tumor-targeting, redox-responsive magnetic and fluorescent PS nanoassembly (NP-RGD), which can be used for localized radiotherapy of tumors guided by NIR/MRI dual-modality imaging. It can efficiently enter tumor cells through αvβ3, producing bright MR contrast for high-resolution imaging of tumor locations. Inside the tumor cell, its breakdown into fluorescent small-molecule products and hydrophobic organic PS not only switches on its NIR fluorescence but also prolongs its retention in the tumor cell, leading to improved photodynamic therapy of tumors (Figure 10b).^168^ In fact, although multimodality imaging overcomes some of the limitations that single imaging has, not all applications require this strategy. Besides, it is noted that it may not be practical to simply add all of the functionality to a single molecule, as the sensitivity of different imaging modalities may vary widely. Finally, the clinical practicality of multimodality imaging reagents remains to be determined, and the vast majority of studies do not appear to have involved clinical patients to characterize the clinically relevant capabilities of the probes.

## 5 Conclusions and Perspectives

Over the past few decades, with rapid advances in nanomaterials science, medicine, and other fields, molecular imaging has evolved from the early days of identifying anatomical changes to the ability to detect specific molecular events. Precise molecular imaging of various modalities relies not only on instrumentation but also on the availability of various types of molecular, supramolecular, and nanostructured imaging reagents. Among them, targeting peptides make imaging reagents based on their constructs interesting by virtue of their properties such as being designable for screening, easily modifiable, and capable of undergoing self-assembly. In this review, we first introduce the history of the development of targeting peptides and the multiple noncovalent bonding interactions involved in their occurrence of self-assembly. Further, we summarize the methods for obtaining targeted peptides, focusing on combinatorial chemical synthesis and screening strategies in detail. Finally, we present application examples of targeting peptides in various imaging modalities.

It is worth noting that although targeting peptides have been widely used in a variety of imaging modalities and have greatly improved imaging sensitivity and resolution, their clinical translation and practical application still face serious challenges. This is mainly in terms of poor in vivo stability susceptibility to enzymatic degradation and rapid renal clearance of the targeting peptide-based imaging agents. Therefore, self-assembly or introduction of non-natural amino acids (D-conformation amino acids, peptidomimetic, etc.) is a possible strategy to solve the above problems. In addition, the coupling of targeting peptides with small-molecule dyes or nanoparticles may change their original properties. Maintaining a sufficient spatial distance between the two components mentioned above is an effective measure, which can be achieved by using spacers or linkers. Finally, in order to meet the needs of physiological environment imaging, a variety of self-assembled peptides have been developed, which can specifically bind to the target and prolong the retention time but also face the difficulty of establishing the structure–effect relationship as well as the difficulty of precise control of the assemblies in vivo. This requires focus on the molecular design of peptides as well as the study of the assembly mechanism. With these ongoing efforts, imaging reagents based on targeting peptides are expected to expand the scope of molecular imaging applications, from understanding the molecular basis of disease to diagnosis, therapeutic regimen design, and outcome assessment, which will ultimately lead to better clinical benefits and personalized medicine.
